# Rare coral under the genomic microscope: timing and relationships among Hawaiian *Montipora*

**DOI:** 10.1186/s12862-019-1476-2

**Published:** 2019-07-24

**Authors:** Regina L. Cunha, Zac H. Forsman, Roy Belderok, Ingrid S. S. Knapp, Rita Castilho, Robert J. Toonen

**Affiliations:** 1 University of Algarve, Campus de Gambelas, 8005-139 Faro, Portugal; 2 Centre of Marine Sciences, CCMAR, University of Algarve, Campus de Gambelas, 8005-139 Faro, Portugal; 3 Hawai‘i Institute of Marine Biology, University of Hawai‘i at Mānoa, Kāne‘ohe, HI 96744 USA

**Keywords:** Across-branch compositional heterogeneity, Introgressive hybridization, Species complex, RADseq, Corals

## Abstract

**Background:**

Evolutionary patterns of scleractinian (stony) corals are difficult to infer given the existence of few diagnostic characters and pervasive phenotypic plasticity. A previous study of Hawaiian *Montipora* (Scleractinia: Acroporidae) based on five partial mitochondrial and two nuclear genes revealed the existence of a species complex, grouping one of the rarest known species (*M. dilatata*, which is listed as Endangered by the International Union for Conservation of Nature - IUCN) with widespread corals of very different colony growth forms (*M. flabellata* and *M*. cf. *turgescens*). These previous results could result from a lack of resolution due to a limited number of markers, compositional heterogeneity or reflect biological processes such as incomplete lineage sorting (ILS) or introgression.

**Results:**

All 13 mitochondrial protein-coding genes from 55 scleractinians (14 lineages from this study) were used to evaluate if a recent origin of the *M. dilatata* species complex or rate heterogeneity could be compromising phylogenetic inference. Rate heterogeneity detected in the mitochondrial data set seems to have no significant impacts on the phylogenies but clearly affects age estimates. Dating analyses show different estimations for the speciation of *M. dilatata* species complex depending on whether taking compositional heterogeneity into account (0.8 [0.05–2.6] Myr) or assuming rate homogeneity (0.4 [0.14–0.75] Myr). Genomic data also provided evidence of introgression among all analysed samples of the complex. RADseq data indicated that *M. capitata* colour morphs may have a genetic basis.

**Conclusions:**

Despite the volume of data (over 60,000 SNPs), phylogenetic relationships within the *M. dilatata* species complex remain unresolved most likely due to a recent origin and ongoing introgression. Species delimitation with genomic data is not concordant with the current taxonomy, which does not reflect the true diversity of this group. Nominal species within the complex are either undergoing a speciation process or represent ecomorphs exhibiting phenotypic polymorphisms.

**Electronic supplementary material:**

The online version of this article (10.1186/s12862-019-1476-2) contains supplementary material, which is available to authorized users.

## Background

Reef-building corals are a complex ecosystem involving biotic interactions within the holobiont (the cnidarian host and its associated microorganisms). Multiple sources of genetic variation involving e.g. parallel evolution [1], and the observed ubiquitous phenotypic plasticity [2] have recognizably obscured phylogenetic inference and species delimitation within the order Scleractinia (stony corals). Defining species boundaries plays a central role in the establishment of conservation policies [3] and biodiversity assessment [4].

The use of a small number of loci usually provides suitable results for distantly related organisms but often produce gene tree discordance when shallower divergences are involved due to population-level effects such as allele frequency changes [5]. However, the concatenation of multiple independent loci can also produce misleading results [6], when, for example, heterogeneity among gene trees is not modeled appropriately [7]. Incomplete lineage sorting (ILS) and interspecific hybridization are among the most common causes of genome-wide heterogeneity in closely related species [8]. Across-branch compositional heterogeneity (i.e., nonstationarity) may also have strong effects on the phylogenetic inference because it groups unrelated taxa that share similar base compositional biases [9–11].

The multispecies coalescent approach [12, 13] and Bayesian species delimitation models using genome-wide data [14] may represent an alternative when single locus are insufficient to solve species boundaries (African beetles, [15]; green algae, [16]). Restriction-site associated DNA sequencing (RADseq) along with the production of thousands of single nucleotide polymorphisms (SNPs) is being increasingly used in shallow systematics, particularly in species delimitation of recent radiations [17, 18] or to evaluate the existence ancestral hybridization and introgression [19].

Phylogenetic inference within stony corals of the order Scleractinia has been hindered by a suit of factors including hybridization [20] and historical introgression [21]. Nuclear internal transcribed spacers (ITS) and a suite of mitochondrial markers have been widely used in coral phylogenies [22–24], but the existence of multiple ITS copies [25, 26] or the reduced utility of mitochondrial markers to delimit species [27, 28] has hindered our understanding of the evolution and molecular ecology of scleractinian corals.

The coral genus *Montipora* (Scleractinia, Acroporidae) is widely distributed across the Indian and the Pacific Oceans [29]. While putative reticulate evolution and hybridization were detected in some Australian *Montipora* species [30], it is not clear to what extent this phenomenon is widespread. A study on the Hawaiian congeners based on several mitochondrial (cytochrome oxidase subunit I, control region, cytochrome *b*, 16S rRNA, and ATP6) and nuclear (ITS and ATPsβ) markers, revealed the existence of a species complex (*M. dilatata*/*M. flabellata*/*M*. cf. *turgescens*), and phylogenetic patterns consistent with ILS, hybridization, polymorphism or phenotypic plasticity but the available data could not resolve among these alternate hypotheses [31].

Our objective was to determine if sampling all 13 nearly complete protein-coding genes of the mitochondrial genome and phylogenomic (RADseq) data would allow the resolution of species boundaries within the *Montipora dilatata*/*M. flabellata*/*M*. cf. *turgescens* complex. Conservation actions should be based on evolutionary distinctiveness metrics (e.g. full-coverage species-level phylogenies diversity over time; [32, 33]). This approach requires a significant amount of data, not always available for the target clade. Other approaches, including expert opinions or phylogenies with lower taxonomic coverage, may also perform well in the establishment of species boundaries [34].

We used the mitogenomic data set to analyse across-branch compositional biases and perform a dating analysis. We included a larger number of scleractinian representatives (six families and 15 genera) with an extensive fossil record required for date estimation. We intended to evaluate if a recent origin of the *M. dilatata* species complex could be hindering phylogenetic inference. RADseq data from 16 samples representing seven nominal *Montipora* species was used to investigate if ILS and/ or introgressive hybridization could be compromising species delimitation within the *M. dilatata* complex. We also evaluate whether the morphological variants of the highly polymorphic congener *M. capitata* can be distinguished at genomic level and how the range of variation within this undisputed species can be compared to their endangered congeners. In addition, a range of phenotypes (laminar, encrusting, plating, and branching) of *M. capitata* and included pooled libraries of distinct colour morphs (red and yellow/orange) previously shown to have different symbiont communities [35] were sampled to characterize within-species variation.

## Results

### Phylogenetic relationships within Scleractinian based on mitogenomic data

Mapping paired reads in Geneious v.8.1.4 to the mitochondrial genome of *Montipora cactus* (GenBank # NC_006902) resulted in a mean of 4583 reads per sample covering 97% of the reference sequence at a mean depth of 61 ± 60 (mean ± standard deviation) per library (Additional file 1: S1B). The concatenated nucleotide dataset of the 55 scleractinians (49 species plus five morphotypes and pooled samples within *M. capitata*) representing 6 families and 15 genera plus the two outgroups (57 taxa in total) comprised 11,484 characters. The net uncorrected p-distance between *M. capitata* and *M. dilatata* species complex was 0.004 ± 0.001. Uncorrected p-distances within the *M. capitata* clade and the *M. dilatata* species complex clade (*M. dilatata*/*M. flabellata*/*M*. cf. *turgescens*) were both zero.

**Fig. 1:**
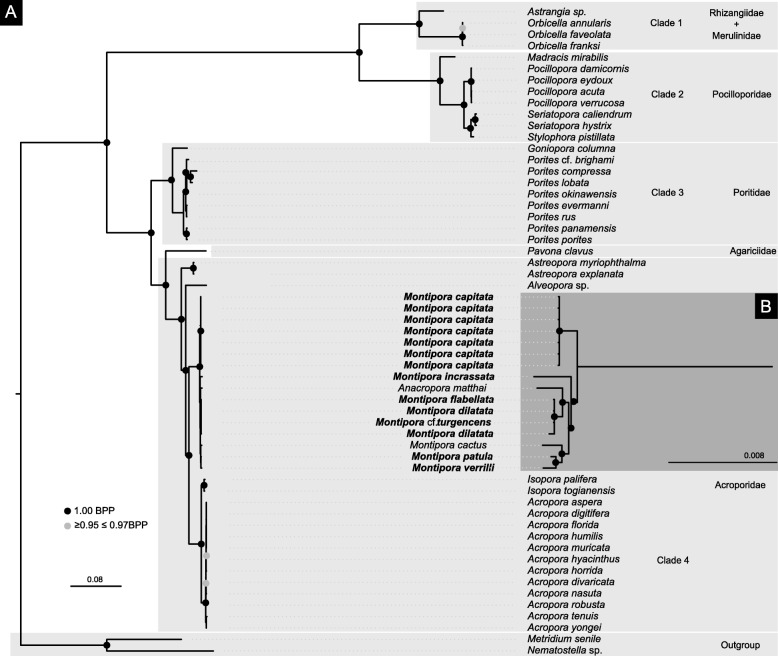
**a** Majority-rule consensus tree of a composition-heterogeneous Bayesian analysis of the concatenated dataset of the 13 protein-coding genes of the mitochondrial genome of 55 scleractinian corals (49 species plus five morphotypes and pooled samples within *M. capitata*) representing 6 families and 15 genera plus the two outgroups *Nematostella* sp. and *Metridium senile*. The NDCH2 analysis was performed in P4 using duplicate runs each consisting of 2 million generations using a GTR + I + Γ model. Bayesian posterior probability values are shown in black circles for values of maximal probability (1.00) and grey circles for values between 0.95 and 0.97. **b** Inset highlighted in dark grey showing a detail of the inferred phylogenetic relationships within *Montipora*. Families are highlighted in light grey. Specimens in bold were sequenced in this study

P4 analysis of the concatenated mitochondrial dataset rejected among-lineage composition homogeneity (*p*-value = 0.00). The analysis under the node-discrete composition heterogeneity model (NDCH2; p-value = 0.85) indicated the existence four main clades including genera from the following families: clade 1 - Rhizangiidae + Merulinidae; clade 2 - Pocilloporidae; clade 3 - Poritidae; clade 4 - Acroporidae (Fig. 1). Similar to previous studies [36, 37], the clade corresponding to the family Acroporidae also included the genus *Alveopora* (Poritidae). Phylogenetic relationships within the *M. dilatata* species complex were unresolved. *Montipora capitata* was retrieved as the sister lineage to the remaining species of the genus. *Montipora patula* and *M. verrilli* grouped together to the exclusion of *M. cactus*. BI analyses performed with MrBayes under the assumption of a homogeneous model of rate change yielded a topology (Additional file 2: S2) identical to P4.

In RAxML, we used the concatenated mitochondrial nucleotide dataset with eight partition schemes as determined by PartitionFinder, and corresponding evolutionary models, all accommodating rate heterogeneity: (1) *nad5*; (2) *nad1*; (3) cyt *b* and *cox3*; (4) *nad2, nad3* and *nad4*; (5) *cox2*, *nad6* and *nad4L*; (6) *atp6*; (7) *atp8* and (8) *cox1*. The best substitution model was GTR + I + Γ for all partitions. This analysis (Additional file 3: S3) yielded a similar topology to the P4 tree with the two following exceptions: (1) phylogenetic relationships within the *Montipora* complex (*M. dilatata*/*M. flabellata*/*M*. cf. *turgescens)* were unresolved in P4 and Mrbayes whereas in RAxML *M. dilatata* (sample Mdil12) from Kane‘ohe bay was retrieved as the sister lineage of all remaining species, and (2) phylogenetic relationships between *M. capitata* morphs were unresolved in MrBayes, while samples 1 and 2 (red plate/branch and red branch) clustered together with high statistical support in RAxML.

### Dating analyses

**Fig. 2:**
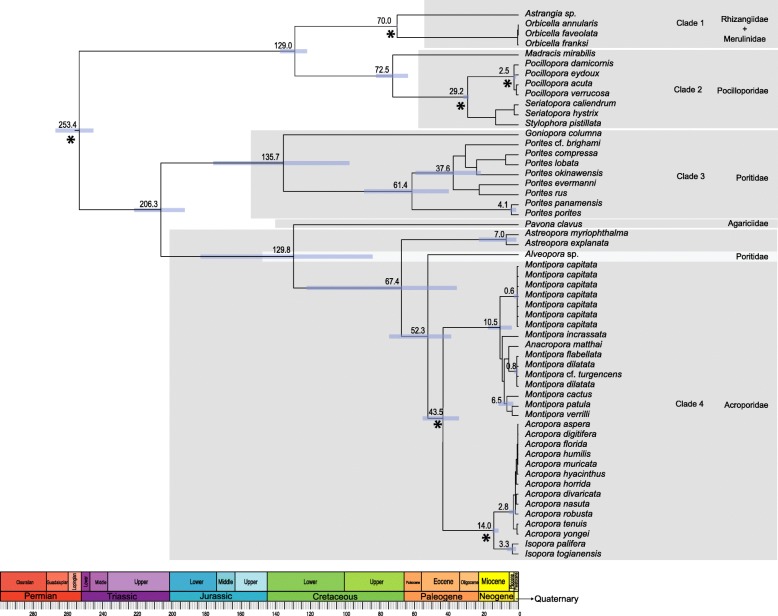
Bayesian divergence dating analysis obtained with Multidivtime. Divergence dates were estimated on the Bayesian topology inferred by P4 and based on the concatenated dataset of the nearly complete 13 mitochondrial protein-coding genes of 55 scleractinian corals (49 species plus five morphotypes and pooled samples within *M. capitata*) representing 6 families and 15 genera plus the two outgroups *Nematostella* sp. and *Metridium senile* (which are automatically removed from the resulting topology). Numbers at the nodes represent age estimates for the main groups in million years. Asteriks at the nodes represent minimum age constraints obtained from the fossil record, and 95% confidence intervals are represented by the blue bars

Multidivtime (Fig. 2) estimate for the crown group age of *Montipora* is 10.5 [5.3–16.6] Myr, while Beast (Additional file 4: S4) shows a more recent origin for the genus at 4.3 [3.6–5.1] Myr. According to Multidivtime, the crown group age of *M. capitata* is 0.6 [0.02–2.4] Myr, whereas Beast estimates it at 0.2 [0.06–0.4] Myr. The crown group age of the complex *Montipora dilatata*/*M. flabellata*/*M*. cf. *turgescens* was estimated at 0.8 [0.05–2.6] Myr or 0.4 [0.1–0.8], as estimated by Multidivtime and Beast, respectively.

### SNP-based analyses

The ‘coral’ data set averaged ~ 946,887 reads passing filtering per library with a coverage depth of 15.63 ± 8.77 (mean ± stdev) (Additional file 1: S1B).

The Venn diagram (Additional file 6: S6) plotted using the program BioVenn [38] shows minimal overlap between putative coral and symbiont loci. 64% (43,423) of the overall loci (67,598) had significant hits to the *M. capitata* draft genome, [39], whereas only 6% (4,286) were significant against the *Symbiodinium minutum* genome [40] and 7% (4,727) against the *Fugacium kawagutii* genome [41]. As such, we used the ‘coral’ data set in all analyses.

The net uncorrected p-distance based on the ‘coral’ data set between *Montipora capitata* and *Montipora dilatata* species complex was 0.301 ± 0.043. Uncorrected p-distances within the *M. capitata* clade and *M. dilatata* species complex clade (*M. dilatata*/*M. flabellata*/*M*. cf. *turgescens*) were 0.056 ± 0.011 and 0.140 ± 0.022, respectively.

**Fig. 3:**
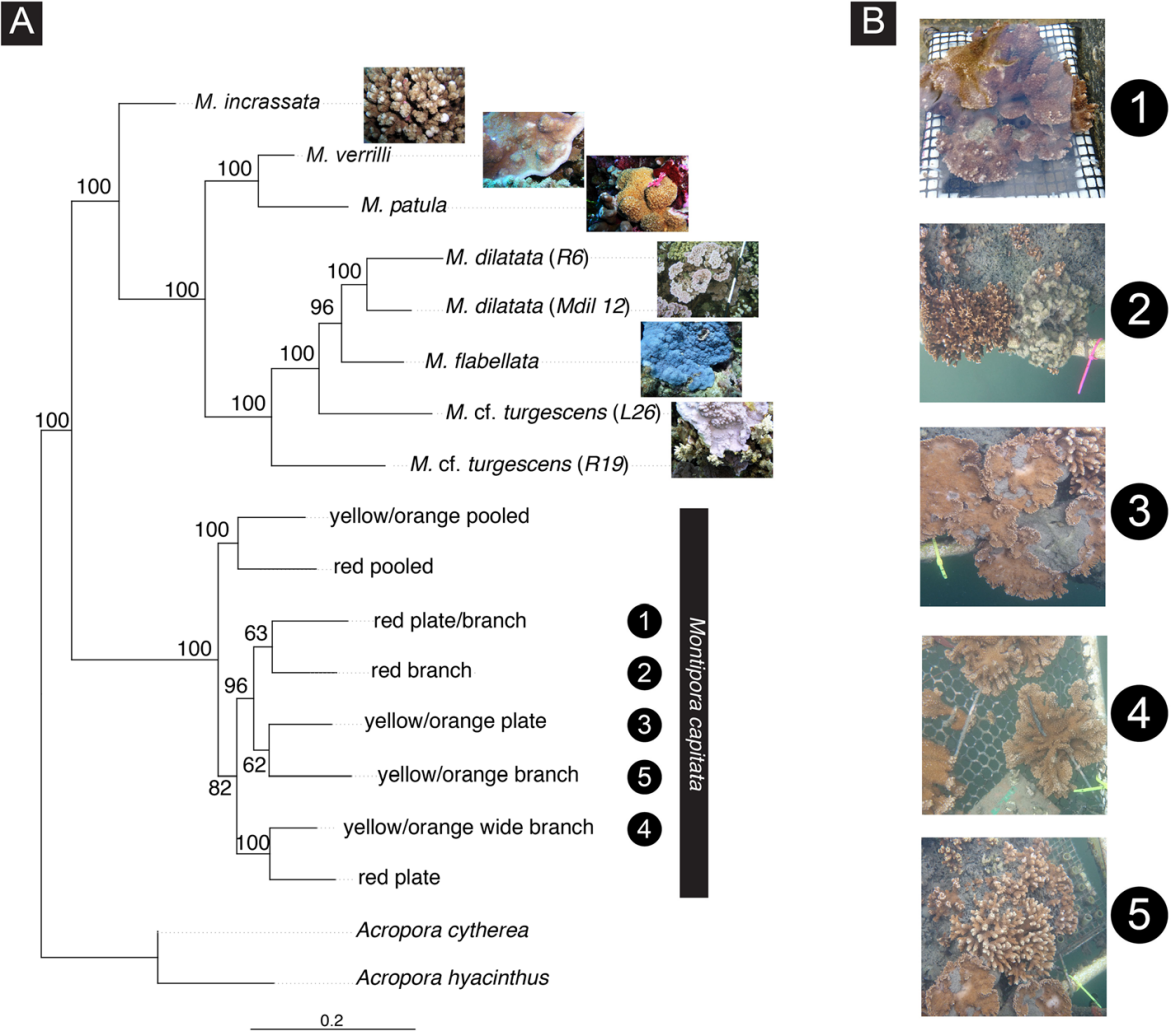
Maximum likelihood phylogram of Hawaiian *Montipora* corals inferred with RAxML based on the ‘coral’ dataset (draft genome-based assembly of RADseq data from 16 samples; 60,602 SNPs). Numbers at the nodes represent Bayesian Posterior Probabilities. *Acropora cytherea* and *A. hyacinthus* are the selected outgroup. Numbers in the black circles, both on the panel images and branches of the phylogenetic tree, correspond to five distinct morphotypes within *Montipora capitata.* Specimen labels are indicated within parentheses

ML analysis based on the ‘coral’ dataset (Fig. 3), recovered the two *M. dilatata* samples in a clade that received high statistical support. *Montipora* cf. *turgescens* (sample L26) did not cluster with its conspecifics (sample R6). *Montipora patula* and *M. verrilli* were retrieved as the sister clade of the species. The ML analysis (Fig. 3) showed some level of correspondence between phenotypic variability and genetic clustering within *Montipora capitata*. Yellow/orange colonies 3 and 5 and red colonies 1 and 2 grouped together but with relatively low statistical support. Images of each species and *Montipora* morphotypes used in the analyses are shown in Additional file 7: S7. We found no genetic association between samples with similar shapes (e.g., colonies 3 and 5 grouped together and show plate and branch shapes, respectively) thus we performed no further analyses on this relationship. More stringent options regarding missing data (−-max-missing 0.75; 16,379 SNPs) yielded an identical ML topology but with less statistical support for the recovered clades (not shown); therefore, we used the file with a larger number of SNPs (60,602 SNPs) in the analyses.

Bayes factor comparisons for species delimitation using MLE (Table 1) clearly reject current taxonomy, favouring the hypothesis that groups *M. capitata* by colour morphs and splits the remaining samples into different species. The second-best hypothesis is the one that considers *M. dilatata* and *M. flabellata* as a single species and splits the remaining samples into different species. The hypothesis that considers the *M. dilatata* complex as a single species received low support.

**Table 1:** Species delimitation using BFD* with SNP data from the Hawaiian *Montipora* based on the ‘coral’ data set. The six analysed models are ranked according to their Bays Factor (BF) value

Model	MLE	Rank	BF
A - current taxonomy	− 572.42	5	N/A
B - splits *M. dilatata* and *M.* cf. *turgescens* samples into different species	−318.88	2	−507.1
C - *M. dilatata* complex as a single species	− 721.67	5	298.5
D - groups *M. capitata* by colour morphs (Yellow/orange; Red)	**− 288.39**	**1**	**− 568.1**
E - Lumps *M. dilatata* into a single species; splits all remaining samples of the complex as different species	−338.36	4	− 468.1
F - Groups *M. dilatata* and *M. flabellata* into a single species; splits *M.* cf. *turgescens*	−328.74	3	− 487.4

*MLE* marginal likelihood estimate, *BF* Bayes factor.

Values in bold correspond to the best estimated model

The species tree based on the ‘coral’ data set estimated by SVDQuartets yielded a topology (Additional file 5: S5) in which *M. dilatata* (samples Mdil12 and R6) groups with *M. flabellata* in the same clade, and the two samples of *M.* cf. *turgescens* do not cluster together. The species tree marginally supports a genetic basis for the observed polymorphisms; only yellow/orange colonies 4 and 5 grouped together (Additional file 5: S5).

### Genomic admixture

Given the conflicting results we obtained regarding phylogenetic relationships within the *M. dilatata* complex depending on the methods used (RAxML: *M. dilatata* monophyletic; SVDQuartets: *M. dilatata* paraphyletic) we tested for the existence of ILS/introgression. We used the D-statistic calculation [42] to distinguish between both processes. Considering the dubious taxonomic status of the species belonging to the complex, we tested for all 30 possible combinations of donor/admixed lineages between the five samples (Table 2).

**Table 2:** Measure of the phylogenetic admixture among the species of the *Montipora dilatata* complex. (*M. dilatata/M. flabellata/M.* cf. *tur*gescens) based on the ‘coral’ data set

Gene tree	Hypotheses	(((P1,P2),P3),O);	ABBA	BABA	D_stat	D_*P*value	introgression
Donor = *Montipora dilatata*: specimen Mdil12	H1	P1 = R6Mdil; P2 = Mflab; P3 = Mdil12; Outgroup = L27incra	247	286	−0.073	0.091	none
	H2	P1 = R6Mdil; P2 = L26turg; P3 = Mdil12; Outgroup = L27incra	151	237	−0.222	**1.3E-05**	13
	H3	P1 = R6Mdil; P2 = R19turg; P3 = Mdil12; Outgroup = L27incra	102	195	−0.313	**6.8E-08**	13
	H4	P1 = L26turg; P2 = R19turg; P3 = Mdil12; Outgroup = L27incra	71	122	−0.264	**2.4E-04**	13
	H5	P1 = Mflab; P2 = R19turg; P3 = Mdil12; Outgroup = L27incra	99	204	−0.347	**1.6E-09**	13
	H6	P1 = Mflab; P2 = L26turg; P3 = Mdil12; Outgroup = L27incra	159	221	−0.163	**1.5E-03**	13
Donor = *M. dilatata*: specimen R6Mdil	H7	P1 = Mdil12; P2 = Mflab; P3 = R6Mdil; Outgroup = L27incra	192	286	−0.197	**1.7E-05**	13
	H8	P1 = Mdil12; P2 = L26turg; P3 = R6Mdil; Outgroup = L27incra	112	237	−0.358	**2.2E-11**	13
	H9	P1 = Mdil12; P2 = R19turg; P3 = R6Mdil; Outgroup = L27incra	82	195	−0.408	**1.1E-11**	13
	H10	P1 = L26turg; P2 = Mflab; P3 = R6Mdil; Outgroup = L27incra	182	166	0.046	0.391	none
	H11	P1 = L26turg; P2 = R19turg; P3 = R6Mdil; Outgroup = L27incra	78	125	−0.232	**0.001**	13
	H12	P1 = Mflab; P2 = R19turg; P3 = R6Mdil; Outgroup = L27incra	109	202	−0.299	**0.000**	13
Donor = *M. flabellata*: specimen Mflab	H13	P1 = Mdil12; P2 = R6Mdil; P3 = Mflab; Outgroup = L27incra	192	247	−0.125	**0.009**	13
	H14	P1 = Mdil12; P2 = R19turg; P3 = Mflab; Outgroup = L27incra	86	204	−0.407	**0.000**	13
	H15	P1 = Mdil12; P2 = L26turg; P3 = Mflab; Outgroup = L27incra	134	221	−0.245	**0.000**	13
	H16	P1 = R6Mdil; P2 = L26turg; P3 = Mflab; Outgroup = L27incra	166	182	−0.046	0.391	none
	H17	P1 = R6Mdil; P2 = R19turg; P3 = Mflab; Outgroup = L27incra	112	202	−0.287	**0.000**	13
	H18	P1 = L26turg; P2 = R19turg; P3 = Mflab; Outgroup = L27incra	70	115	−0.243	**0.001**	13
Donor = *M.* cf. *turgescens*: specimen R19turg	H19	P1 = Mdil12; P2 = R6Mdil; P3 = R19turg; Outgroup = L27incra	82	102	−0.109	0.140	none
	H20	P1 = Mdil12; P2 = L26turg; P3 = R19turg; Outgroup = L27incra	84	71	0.084	0.296	none
	H21	P1 = Mdil12; P2 = Mflab; P3 = R19turg; Outgroup = L27incra	86	99	−0.070	0.339	none
	H22	P1 = R6Mdil; P2 = Mflab; P3 = R19turg; Outgroup = L27incra	112	109	0.014	0.840	none
	H23	P1 = R6Mdil; P2 = L26turg; P3 = R19turg; Outgroup = L27incra	92	78	0.082	0.283	none
	H24	P1 = L26turg; P2 = Mflab; P3 = R19turg; Outgroup = L27incra	70	85	−0.097	0.228	none
Donor = *M.* cf. *turgescens*: specimen L26turg	H25	P1 = Mdil12; P2 = R6Mdil; P3 = L26turg; Outgroup = L27incra	112	151	−0.148	0.016	none
	H26	P1 = Mdil12; P2 = R19turg; P3 = L26turg; Outgroup = L27incra	84	122	−0.184	**0.008**	13
	H27	P1 = Mdil12; P2 = Mflab; P3 = L26turg; Outgroup = L27incra	134	159	−0.085	0.144	none
	H28	P1 = R6Mdil; P2 = Mflab; P3 = L26turg; Outgroup = L27incra	166	166	0.000	1.000	none
	H29	P1 = R6Mdil; P2 = R19turg; P3 = L26turg; Outgroup = L27incra	92	125	−0.152	0.025	none
	H30	P1 = R19turg; P2 = Mflab; P3 = L26turg; Outgroup = L27incra	115	85	0.150	0.034	none

alpha = 0.01.

Significant *P*-values showing evidence of introgression are shown in bold

Results showed evidence of introgression because of the significant discordant ABBA/BABA site patterns (highlighted in bold in Table 2) between: (i) the donor population *M. dilatata* (sample Mdil12) vs. all samples but *M*. cf. *turgescens* (sample R19turg); (ii) the donor lineage *M. dilatata* (sample R6) vs. all samples but *M*. cf. *turgescens* (sample R19turg); (iii) the donor lineage *M. flabellata* vs. all samples but *M*. cf. *turgescens* (sample R19turg), and (iv) the donor lineage *M*. cf. *turgescens* (sample L26turg) vs. *M. dilatata* (sample Mdil12).

## Discussion

Phylogenetic reconstructions and species delimitation of scleractinian corals have long been hampered by a disagreement between genetic data and colony-level morphology [43–47]. This challenge is particularly difficult among genera in which colony morphology is known to be highly variable, such as the Hawaiian *Montipora*. For example, the rare coral *M. dilatata* was listed as Endangered by IUCN following a 1996 bleaching event that reduced the population to just two known colonies in the Main Hawaiian Islands [48]. In 2009, *M. dilatata*, *M. flabellata, M. turgescens, M. patula* and *M. verrilli* were included in a petition to list 83 coral species for protection under the US Endangered Species Act [49]. Several genetic markers called into question the taxonomic validity of these species, however there was not enough data to test alternative hypotheses of possible introgression, incomplete lineage sorting or rapid speciation [31].

Here we used all 13 mitochondrial protein-coding genes and reduced representation genomic sequencing in excess of 60,000 SNPs to evaluate phylogenetic relationships within the Hawaiian *M. dilatata* species complex in a more rigorous hypotheses testing framework than the [31] study.

### *Montipora* phylogenetic relationships sampling greater portions of the genome

Overall, our phylogenetic reconstructions based on mitogenomic data are consistent with previous work, particularly when examining deeper nodes but show some discrepancies, mostly at intra-generic level. BI and ML analyses (Fig. 1, Additional file 2: S2 and Additional file 3: S3) showed that *Acropora* and *Isopora* are sister genera to *Montipora*, as reported in previous studies using a more comprehensive taxon sampling of the Scleractinia [50, 51]. The inclusion of *Anacropora matthai* within the *Montipora* clade (Fig. 1, Additional file 2: S2 and Additional file 3: S3) was also already described in [36, 52, 53], suggesting that its taxonomic status needs a revision. Our mitochondrial-based analyses (Fig. 1) further confirmed lack of differentiation between *Porites lobata* and *P. compressa* as described in [54].

All analyses based on genomic data (with the single exception of the RAxML topology) revealed that *M. dilatata* and *M. flabellata* correspond to a single species, and the two samples of *M.* cf. *turgescens* represent different evolutionary significant units, which does not reflect current taxonomy. Additional population level sampling is needed to determine if the genetic structure within this clade is due to partitioning across geographic regions, habitats, or morphology. Results presented here show the existence of hidden diversity within the genus *Montipora* that was not detected by the mitochondrial markers, showing the importance of sampling a greater portion of the genome for species delimitation.

### Impact of rate heterogeneity on phylogenetic inference and dating estimates

The use of models that do not consider lineage-based compositional heterogeneity may provide strong support for an incorrect topology (e.g., wrong placement of eukaryotes [55] or the placental mammals [56] in the tree of life). As such, we explored if there were topological differences between the analyses based on mitogenomic data performed under a homogeneous model of sequence evolution or accommodating among-lineage compositional heterogeneity.

Although our results showed the existence of heterogeneity in the concatenated mitochondrial dataset (the homogeneous composition vector was rejected, *P* = 0.000), its impact on the scleractinian phylogeny seems minimal given the similar topologies obtained with methods having different assumptions (RAxML: gamma model of rate heterogeneity, P4: across-branch compositional heterogeneity vs. MrBayes: rate homogeneity). On the other hand, our results show that heterogeneity in among-lineage base composition clearly affects dating analyses. Beast (rate homogeneity) showed more recent age estimates at the tips and an older root (Additional file 4: S4) when compared with Multidivtime (rate heterogeneity; Fig. 2). The crown group ages of the Hawaiian *Montipora* were quite different if estimated with Multidivtime (10.5 Myr) or Beast (4.3 Myr). Only a few studies are taking into account among-lineage compositional biases when dating divergences (e.g., cichlids [57]), and usually involve the removal of problematic genes and loss of significant phylogenetic information. In the methodology we used, age estimates are based on the complete data set and performed over a tree that was inferred with nonstationary models.

### Genetic association with phenotypic variability

We used phenotypic variation among well-characterized colour variants of *M. capitata* to evaluate if there is a genetic basis for the observed polymorphisms. Bayes Factor Delimitation (BFD*) based on > 60,000 SNPs showed a significant correlation between colour variability and genetic clustering, as indicated by the highest BF value of the model that included the grouping of samples according to their colour (Hypothesis D; Table 1).

ML based on the ‘coral’ data set grouped *M. capitata* colonies by colour (Fig. 3), suggesting that there may be a genetic component to these variants, warranting further attention. Red and yellow/orange colonies of *M. capitata* growing under identical conditions revealed distinct fluorescent phenotypes [58], which may also support a genetic basis underlying the colour polymorphism. Furthermore, Innis and colleagues [35] found that *M. capitata* colour morphs contain distinct proportions of clades of photosynthetic dinoflagellates family *Symbiodiniaceae,* in the genera *Cladocopium* and *Durusdinium* (formerly clade C and D), and Shore-Maggio and colleagues showed the same was true of the microbial communities [59] and disease susceptibility [60] of these colour morphs. We found no genetic basis for the phenotypic differences associated to colony shape (Table 1).

### Incomplete lineage sorting, introgressive hybridization or a recent origin for the Hawaiian *Montipora*?

The difficulty in establishing species boundaries within the *M. dilatata* complex has been attributed to either ILS [31] or interspecific hybridization [30], but these assumptions were not previously tested. D-statistics tests (Table 2) identified the existence of introgression between all samples of the complex. However, only *M. dilatata*, *M. flabellata* and sample L26turg (*M.* cf. *turgescens*) were identified as donor lineages. Genomic admixture was identified between sympatric lineages (e.g., *M. dilatata* - sample Mdil12 vs. *M. flabellata* both from Kāne‘ohe Bay, O‘ahu, Hawaii), but also among samples found in allopatry (e.g., *M.* cf. *turgescens* - sample L26turg, Kure Atoll, Northwestern Hawaiian Islands, NWHI vs. *M. dilatata* - sample Mdil12, Kāne‘ohe Bay).

Reproductive isolation between species is a gradual process allowing introgression of allopatric lineages upon secondary contact, long time after their divergence [61]. In broadcast spawners like *Montipora* corals, which have the ability to disperse over long distances and maintain gene flow among distant populations, divergence may take even longer and we estimated a very recent origin for the species complex (see next paragraph). Further, the geographic distribution of the nominal species of the complex largely overlaps, and introgression in parapatric lineages was recently described [62]. Introgressive hybridization was also detected in another group of scleractinian corals of the genus *Pocillopora* [63].

Phylogenetic inference between closely related taxa can be hindered by several factors such as lack of genetic variation in recently-derived taxa [64]. Our dating analysis estimated the crown group age of the complex *Montipora dilatata*/*M. flabellata*/*M*. cf. *turgescens* under the NDCH2 composition heterogeneity model at 0.8 Myr [0.04–2.62] (Fig. 2). This recent origin is most certainly hampering phylogenetic inference.

## Conclusions

Delineating species is critical to our ability to address conservation and management goals, but the boundaries between species can be challenging in groups such as scleractinian corals because genetic data disagree with established taxonomy and gross morphology. Here, we included all 13 mitochondrial coding-genes and RADseq data (> 60,000 SNPs) to test how previous results could be impacted by lack of informative loci, across-branch compositional heterogeneity or biological processes such as introgressive hybridization. We generated the most comprehensive mitogenomic data set gathered to date for scleractinian corals. Despite the volume of data available, we still fail to clearly resolve relationships among nominal species within the *M. dilatata* species complex. Nevertheless, genomic data revealed new findings: (1) current taxonomy [65] does not reflect the true diversity within the genus; (2) species delimitation tests favoured the model that considered *M. dilatata* and *M. flabellata* as a single species and splits the two samples of *M*. cf. *turgescens* as different evolutionary significant units; (3) species delimitation tests and ML analysis supported a genetic basis for the observed colour polymorphisms in *M. capitata*, and (4) the existence of introgression among the species of the complex is confirmed. Dating analyses indicated a very recent origin for the complex. Age estimates varied depending on whether compositional heterogeneity was taken into account (0.8 Myr) or rate homogeneity was assumed (0.4 Myr). Genomic admixture was identified between sympatric lineages but also between samples found in allopatry; however, the geographic distribution of the nominal species belonging to the complex largely overlaps, allowing parapatric introgression. Phylogenomic data presented here questions the endangered status of *M. dilatata* and the taxonomic validity of the remaining species of the complex also showing the existence of cryptic genetic diversity within the genus warranting further study*.*

## Methods

### Taxon sampling

Tissue from 16 samples belonging to the genus *Montipora* were collected from Hawai’i and the Central Pacific between 2010 and 2013 and stored either in salt-saturated dimethyl sulfoxide (DMSO) buffer [66] or in 95% ethanol prior DNA extraction. All our samples had a mean sample size of 1–2 cm^2^ and were taken by non-lethal tissue biopsy of the corals as required by the State collection permits (SAP-HIMB2010, SAP-HIMB2011, SAP-HIMB2012, SAP-HIMB2013) that we obtained to sample these organisms. There were no colonies harvested (or permanently damaged) for this research (all heal within less than 30 days from the biopsy sampling), and all were sampled as approved by the State and Federal agencies responsible for their management.

The samples included six different morphological types (morphs) and two pooled samples (red and yellow/orange) of *M. capitata*, two samples of *M. dilatata*, two samples of *M. turgescens*, and one sample from each of the following nominal species: *M. verrilli*, *M. patula, M. incrassata*, and *M. flabellata* (Additional file 1: S1A). We sampled a range of variation within *M. capitata* including samples (five fragments from each colour morph) that were collected around the Hawai‘i Institute of Marine Biology and grown from ~2cm fragments in a ‘common garden’ floating rack, in a lagoon environment for six years.

For the mitogenomic analyses, we used all 13 mitochondrial protein-coding genes from 14 *Montipora* samples sequenced in this study and retrieved from the GeneBank 41 coral species of the order Scleractinia, representing a total of 6 families and 15 genera (GeneBank accession numbers in the Additional file 1: S1A, Genomic libraries were deposited in the NCBI short read archive with the bioproject ID PRJNA554733). Taxon sampling essentially focused on the family Acroporidae (genera *Acropora*, *Montipora*, *Isopora* and *Anacropora*). The remaining taxa were selected because of their fossil record, which is required for calibration in the dating analysis.

### Laboratory procedures, sequence alignment and genetic distances

Genomic DNA was extracted from coral tissue using the OMEGA (BIO-TEK) E-Z 96 Tissue DNA Kit and eluted in 2 × 100 μl. Extractions were quantified using the AccuBlueTM (Biotium, Inc.). Libraries were generated using the ezRAD method [67, 68]. Genomic DNA was digested using the isoschizomer restriction enzymes *Mbo*I and *Sau*3AI (New England BioLab), which both cleave at GATC recognition sites. Details on library preparation are described in Johnston [63]. All libraries were size-selected between 300 and 500 bp and only after passing the quality control steps (bioanalyser and qPCR) were sequenced at the Hawai’i Institute of Marine Biology (HIMB) Genetics Core Facility following the Illumina TruSeq Sample Prep v2 Low Throughput protocol.

The 16 Illumina *Montipora* libraries were assembled to the mitochondrial genome of *Montipora cactus* (NC_006902) to get the consensus sequences using the default settings (high sensitivity iterated up to five times and the medium/read mapping settings) in GENEIOUS v.8.1.4 (Biomatters, Auckland, New Zealand; https://www.geneious.com). We failed to get reliable mitochondrial consensus sequences from two of the sequenced libraries corresponding to the samples MturgL26 (*M.* cf. *turgescens*) and mCAPsamp (*M. capitata*).

Following Medina et al. [69], the actinarians *Nematostella* sp. and *Metridium senile* (GenBank accession numbers: DQ643835 and HG423143, respectively) were selected as the outgroup of the Scleractinia used in the mitogenomic analyses. Each mitochondrial protein-coding gene from the 55 scleractinians (14 samples from this study) plus the two outgroups (57 taxa in total) was individually aligned with Mafft v7.245 [70]. The invertebrate mitochondrial genetic code was used to detect open reading frames (ORFs) and stop codons and deduce the amino acid sequences of each of the 13 partial mitochondrial protein-coding genes in Mesquite v3.2 [71]. Those alignments were concatenated into a single dataset (57 taxa, 11,484 bp) used in Bayesian inference (BI) and maximum likelihood (ML) analyses. All newly generated mitochondrial protein-coding sequences have been deposited in GenBank, for which accession numbers are provided in the Additional file 1: S1A.

Sequence distances of the mitochondrial concatenated data set with 500 bootstrap replicates were calculated in Mega 5 [72]. Uncorrected within p-distances were computed for the following groups: (1) all *M. capitata* samples representing a wide range of colony morphology, and (2) all samples within the *M. dilatata* complex (*M. dilatata:* Mdil12 + R6; *M.* cf. *turgescens*: R19 + L26 and *M. flabellata*). Net-sequence distances were estimated between *M. capitata* and *M. dilatata* complex.

### Phylogenetic analyses of mitochondrial DNA based on standard homogeneous models

Bayesian Inference (BI) analyses based on the concatenated mitochondrial dataset (57 taxa, 11,484 bp) were performed with MrBayes v3.2.1 [73] under a homogeneous model of rate change (rates = equal). All analyses were run for 9 × 10^7^ generations (four simultaneous Markov chains; 1 × 10^3^ sample frequency) following a discarded burn-in of 10%. The convergence to the stationary distributions was confirmed by inspection of the MCMC samples using Tracer v. 1.6 [74].

### Across-branch compositional heterogeneity

To analyse if there was across-branch compositional heterogeneity in the concatenated nucleotide data set from the 13 mitochondrial protein-coding genes representing 55 Scleractinian lineages, we first inferred a phylogeny under a model of composition homogeneity as implemented in P4 v1.2.0 [9]. As the results indicated a null probability for this model we used the node-discrete composition heterogeneity (NDCH2) model [9] with GTR + I + Γ, also implemented in P4 v1.2.0. This software performs a Bayesian MCMC analysis which allows composition to vary among lineages, with a distinct composition vector for each node [11].

We performed ML analysis of the concatenated mitochondrial data set with RAxML v8.2.10 [75] using the option –q that specifies the file name which contains the assignment of models to alignment partitions for multiple models of substitution under the GTR-CAT approximation (gamma model of rate heterogeneity). We used PartitionFinder2 v.2.1.1 [76] with the corrected Akaike Information Criterion (AICc) to select the best partitioning scheme and corresponding best-fit model of substitution. The best-scoring ML tree was determined from 100 randomized maximum-parsimony starting trees using the rapid hill-climbing algorithm, and 1000 bootstrap replicates were drawn on each best-scored ML tree using the exhaustive bootstrap algorithm.

P4, BI and ML analyses were performed on the CCMAR computational cluster facility (http://gyra.ualg.pt) and on the R2C2 computational cluster facility provided by the IT Department of the University of Algarve.

### Dating analyses of mitogenomic data

To date major splitting events within Hawaiian *Montipora* and estimate the origin of the *M. dilatata* complex, we used 55 Scleractinian lineages that exhibit extensive fossil record and have mitogenomic data available. Two relaxed molecular-clock approaches were used to evaluate the impact of rate heterogeneity on date estimates. The software Multidivtime [77] includes an input topology, which in this case was set to the one inferred by P4 that accommodates across-branch compositional heterogeneity, and Beast v.1.8.4 [78] allows the use of rate homogeneity models.

Multidivtime: following Thorne and Kishino [79], we used PAML v.3.14 [80] to estimate ML parameters using a discrete gamma distribution with five rate categories and the F84 model of nucleotide substitution that was selected because of computational tractability [81]. Estbranches [82] was used to estimate branch lengths and subsequently Multidivtime was used to estimate divergence times. This method requires a prior assumption for the mean and standard deviation of the time of the ingroup root node (rttm) that represents the calibration of the root of the tree. This parameter was set to 24.5 time units, where in this analysis, one-time unit represents 10 million years (Myr). This value was based on the earliest record of the true scleractinians in during the Anisian (≈ 245) Myr [83, 84]. The standard deviation of the prior distribution was set to its maximum value (equal to the mean) to avoid violation of the definition of a prior. Calibrations are indicated in Table 3. The MCMC method was employed to approximate both prior and posterior distributions [82]. Initial parameter values were randomly selected to initialize the Markov chain and then, a burn-in period of 100,000 cycles was completed before parameters were sampled from the MCMC chain. Afterwards, the state of the Markov chain was sampled every 100 cycles until a total of 10,000 generations.

**Table 3:** Calibration points used in the mitogenomic dating analysis

	Calibration points (in million years)	Description	References
1	[70.1–69.9]	Divergence between *Astrangia* and *Montastraea*	Medina et al., 2006 [69]; Park et al., 2012 [100]; Veron, 1995 [101]
2	[56.0–33.9]	Divergence between *Montipora* and *Acropora*	Wells, 1956 [102]
3	[42.7–28.4]	Divergence between *Pocillopora* and *Seriatopora*	Simpson et al., 2011 [84] ; Strauss and Sadler, 1989 [103]
4	[5.0–0.0]	The origin of *Porites porites*	Budd and Jonhson, 1999 [104]
5	[15.0–5.3]	Divergence between *Isopora* and *Acropora*	Simpson et al., 2011 [84]; Strauss and Sadler, 1989 [103]
6	[0.99–2.99]	Estimated age for the genus *Pocillopora*	Jonhston et al., 2017 [63]

Beast: we selected the Birth-Death Incomplete Sampling tree prior because we were only using a fraction of the extant Scleractinia. As we wanted to compare date estimates between models incorporating across-branch compositional heterogeneity as implemented in P4/Multidivtime and homogeneous models, we used Beast without the gamma model. We used the same calibrations as in the Multidivtime analysis described in Table 3 that were modeled with a normal distribution: (1) divergence between *Astrangia* and *Montastraea* (mean = 70, stdev = 0.1, 2) divergence between *Montipora* and *Acropora* (mean = 44.95, stdev = 11.05, 3) divergence between *Pocillopora* and *Seriatopora* (mean = 35.55, stdev = 7.15, 4) estimated origin of *Porites porites* (mean = 2.5; stdev = 2.5, 5) divergence between *Isopora* and *Acropora* (mean = 10.15, stdev = 4.85, 6) estimated age for the genus *Pocillopora* (mean = 1.99, stdev = 0.65). We ran two independent runs for 100,000,000 generations, sampling every 1000 generations. The final tree was produced by TreeAnnotator using the “maximum clade creditability” option and mean node height, after burn-in of 10% of the generations. The convergence to the stationary distribution was confirmed by inspection of the Markov Chain Monte Carlo (MCMC) samples and of effective sample sizes (ESS should be > 200) using Tracer v1.6.0 [74].

### SNP-based analyses

We used 16 individuals representing a range of morphologies and two pooled samples representing common colour morphs (yellow/orange and red) of *M. capitata,* to characterize within species variability within this notoriously polymorphic species and determine possible relationships between genotype and phenotype (more details in Additional file 1: S1A). In addition, we included two samples of each nominal species *M. dilatata* and *M.* cf. *turgescens*, and one sample from *M. flabellata* to analyse phylogenetic relationships within the *M. dilatata* species complex. We also included a sample of *M. incrassata* and *M. verrilli* to further analyse phylogenetic relationships within *Montipora*.

FastQC was used for a preliminary quality control of the pair-end reads [85]. The raw reads from Illumina were cleaned with Trim Gallore! [86] and subsequently analysed with dDocent 2.2.25 [87, 88], a pipeline that combines several software packages (https://github.com/jpuritz/dDocent/). All libraries were mapped to the *M. capitata* draft genome [39] using BWA [89] with the following settings: -L 20,5 -t 32 -a -M -T 10 -A 1 -B 4 -O 6 -R. We used vcftools v0.1.15 [90] to filter the resulting variant call file (VCF) from dDocent analysis using the following options: --remove-indels --max-missing 0.50 --thin 300 --mac 3 --minQ 30. We performed an additional analysis using max-missing = 0.75 to evaluate the impact of missing data on phylogenomic reconstruction.

To evaluate the level of contamination from *Symbiodiniaceae de-novo* assembly of all data was performed with dDocent v2.2.25 [87, 88] with a clustering threshold of 0.85, default mapping and SNP calling parameters. Prior to filtering, only loci with at least 2x coverage within libraries and present in at least 3 libraries were retained. The reference sequences for all loci (*n* = 67,598) generated by dDocent were then compared at the amino acid-level using tBLASTx [91] to several available reference genomes in order to determine if the locus was of putative coral or *Symbiodiniaceae* origin.

To convert the filtered SNPs in VCF format to PHYLIP for phylogenetic analysis we used a python script (https://github.com/edgardomortiz/vcf2phylip) that also performs an additional filtering (a minimum of 10 samples are required to be present at a locus, where the default value is 4 to use in a phylogenomic analysis). The dataset hereafter called ‘coral’ consisted of 60,602 SNPs.

Uncorrected p-distances with 500 bootstrap replicates were calculated in Mega 5 [72] following the same procedure described for the mitochondrial data.

ML analyses based on the ‘coral’ data set were performed with RAxML v8.2.10 [75]. Following the manual recommendations, the analysis was run under the GTRGAMMA evolutionary model. The best-scoring ML tree was determined from 100 randomized maximum-parsimony starting trees using the rapid hill-climbing algorithm, and 100 bootstrap replicates were drawn on the best-scored ML tree using the exhaustive bootstrap algorithm. *Acropora cytherea* was selected as outgroup.

We used Bayes delimitation with genomic data (BFD*), a species delimitation method for analysis of SNP data [14] to establish the number of species within *Montipora*. BFD* combines the Beast v.2.4.8 [92] add-on SNAPP [93] with path sampling that estimates marginal likelihoods to use in Bayes factor model selection [14]. We used the VCF file from the dDocent run based on the *M. capitata* draft genome assembly. The filtered VCF (as described earlier) was converted to a binary nexus format using the script vcf2phylip (https://github.com/edgardomortiz/vcf2phylip; [94]). We conducted path sampling with 24 steps to estimate the marginal likelihood estimates (MLE). The MCMC chain for path sampling analysis was run in Beast v.2.4.8 for 1,000,000 generations and sampled every 1000 with a pre-burnin of 100,000. We used the framework of Kass and Raftery [95] to evaluate the strength of support of Bayes factor (BF) comparisons of competing models. A positive BF test statistic (2 x log_e_) reflects evidence in favor model 1, whereas negative BF values support model 2 [14]. A BF value between 2 and 6 represents positive evidence and BF > 10 is decisive. We tested six different hypotheses: (A) supports current taxonomy (*M. verrilli, M. patula, M. capitata, M. incrassata, M. flabellata, M.* cf. *turgescens* and *M. dilatata*) with all individuals of the same nominal species grouping together; (B) splits *M.* cf. *turgescens* and *M. dilatata* samples in different species; (C) groups all samples belonging to the *Montipora dilatata* complex into a single species: *M.* cf. *turgescens* (samples L26 + R19); *M. flabellata*; *M. dilatata*, (samples R6 + Mdil12); (D) groups *M. capitata* samples by colour morph (red and yellow/orange) as a single species, and the remaining *Montipora* samples as separate species; (E) lumps *M. dilatata* into a single species; splits all remaining samples of the complex as different species; (F) groups *M. dilatata* and *M. flabellata* into a single species; splits *M.* cf. *turgescens*. *Acropora cytherea* was used as outgroup in all models.

We used SVDQuartets (Singular Value Decomposition for quartets) [96] to estimate the species tree under the multi-species-coalescent model from SNP data (‘coral’ data set), as implemented in PAUP* v.4.0a (build 163) [97]. We assessed node support with 100 bootstraps, and an exhaustive search of all possible quartets (3060) were evaluated using the QFM quartet assembly algorithm.

### Testing introgression

Phylogenetic discordances (differences in gene tree topologies) can result from either incomplete lineage sorting (ILS) or introgressive hybridization. Given the conflicting patterns of the ML SNP-based topology based and the species tree, we tested all 30 possible combinations of genomic admixture among the nominal species belonging to the species complex *M. dilatata*/*M. flabellata/ M.* cf. *turgescens* (see Table 2 for further details). We selected *M. incrassata* as outgroup based on the results we obtained in the phylogenetic analyses.

We estimated the Patterson’s D-statistic [98, 99] running the python script dfoil.py https://github.com/jbpease/dfoil with the “--mode dstat” option for a four-taxon D-statistic calculation [42]. In a four-taxon phylogenetic relationship represented by (((P1, P2), P3), O) where P represents the lineages of interest for hybridization and O the outgroup, the D-statistic compares two incongruent SNP patterns, ABBA and BABA, in which “B” is the derived allele and “A” the ancestral allele [98, 99]. In the absence of gene flow and random mating, frequencies of the two patterns are expected to be similar [99]. Under a ILS scenario, we would expect that both patterns (ABBA/BABA) would be sampled with equal frequency while a statistically significant imbalance would reflect introgression [42].

## Additional files

Additional file 1: **S1.** A. Species_list: Scleractinian corals used in the mitogenomic data set, accession numbers corresponding to the complete mitochondrial genomes retrieved from GenBank and to the newly sequenced 13 partial mitochondrial protein-coding genes of the 14 *Montipora* samples used in the mitogenomic analyses. List of *Montipora* samples used in the RADseq analyses. B. Sample_information: collected sample information regarding mitogenomic and genomic analyses (number of reads, reads length, mean depth coverage and standard deviation, percentage of the reference sequence). (XLSX 18 kb)

Additional file 2: **S2.** A. Bayesian analysis showing phylogenetic relationships among 55 scleractinian corals (49 species plus five morphotypes and pooled samples within *M. capitata*) representing 6 families and 15 genera plus the two outgroups *Nematostella* sp. and *Metridium senile* based on the concatenated mitochondrial dataset (13 protein-coding genes). B. Inset showing a detail of the topology referring to the genus *Montipora* zoomed 20x. This analysis was produced by MrBayes under a homogeneous model of rate change. Bayesian posterior probability (BPP) values are shown in black circles for values of maximal probability (1.00) and a grey circle for a value of 0.57. (JPG 587 kb)

Additional file 3: **S3.** A. *Maximum* likelihood phylogram of 55 scleractinian corals (49 species plus five morphotypes and pooled samples within *M. capitata*) representing 6 families and 15 genera plus the two outgroups *Nematostella* sp. and *Metridium senile* based on the concatenated mitochondrial dataset (13 protein-coding genes) produced by RAxML under a gamma model of rate heterogeneity. B. Inset showing a detail of the topology referring to the genus *Montipora* zoomed 100x. Numbers at the nodes represent bootstrap proportions. (PDF 230 kb)

Additional file 4: **S4.** Beast maximum clade credibility chronogram showing main cladogenetic events among 55 scleractinian corals (49 species plus five morphotypes and pooled samples within *M. capitata*) representing 6 families and 15 genera plus the two outgroups *Nematostella* sp. and *Metridium senile*. The 95% highest posterior density (HPD) intervals are represented by the blue bars, and numbers at the nodes represent million years. (PDF 226 kb)

Additional file 5: **S5.** Species tree based on 60,602 SNPs from the ‘coral’ data set estimated with SVDquartets. Numbers at the nodes represent bootstrap proportions. Image plates represent *in-situ* photographs of *Montipora* species sampled for this study and of the morphotypes within *Montipora capitata*. (JPG 386 kb)

Additional file 6: **S6.** Venn diagram showing the overlap between putative coral (*Montipora capitata*) and symbionts (*Symbiodinium minutum* and *Fugacium kawagutii*) loci. (PDF 109 kb)

Additional file 7: **S7.** In-situ photographs of *Montipora* species sampled for this study and of the morphotypes within *Montipora capitata*. (JPG 3488 kb)

## Data Availability

The datasets generated and/or analysed during the current study are available in the GeneBank (accession numbers in the Additional file 1: S1A and NCBI SRA bioproject ID PRJNA554733).

## Abbreviations

BFD*: Bayes Factor Delimitation with genomic data
DMSO: Salt-saturated dimethyl sulfoxide
HIMB: Hawai’i Institute of Marine Biology
ILS: Incomplete lineage sorting
ITS: Nuclear internal transcribed spacers
IUCN: International Union for Conservation of Nature
NCBI: National Center for Biotechnology Information
NDCH2: Node-discrete composition heterogeneity model
NWHI: Northwestern Hawaiian Islands
RADseq: Restriction-site associated DNA sequencing
SNPs: Single nucleotide polymorphisms
VCF: Variant call file
