# Supplementary figures and images for: Rare coral under the genomic microscope: timing and relationships among Hawaiian Montipora
Source: BMC Evol Biol. 2019 Jul 24;19:153. doi: 10.1186/s12862-019-1476-2 (PMC6657087; doi:10.1186/s12862-019-1476-2)

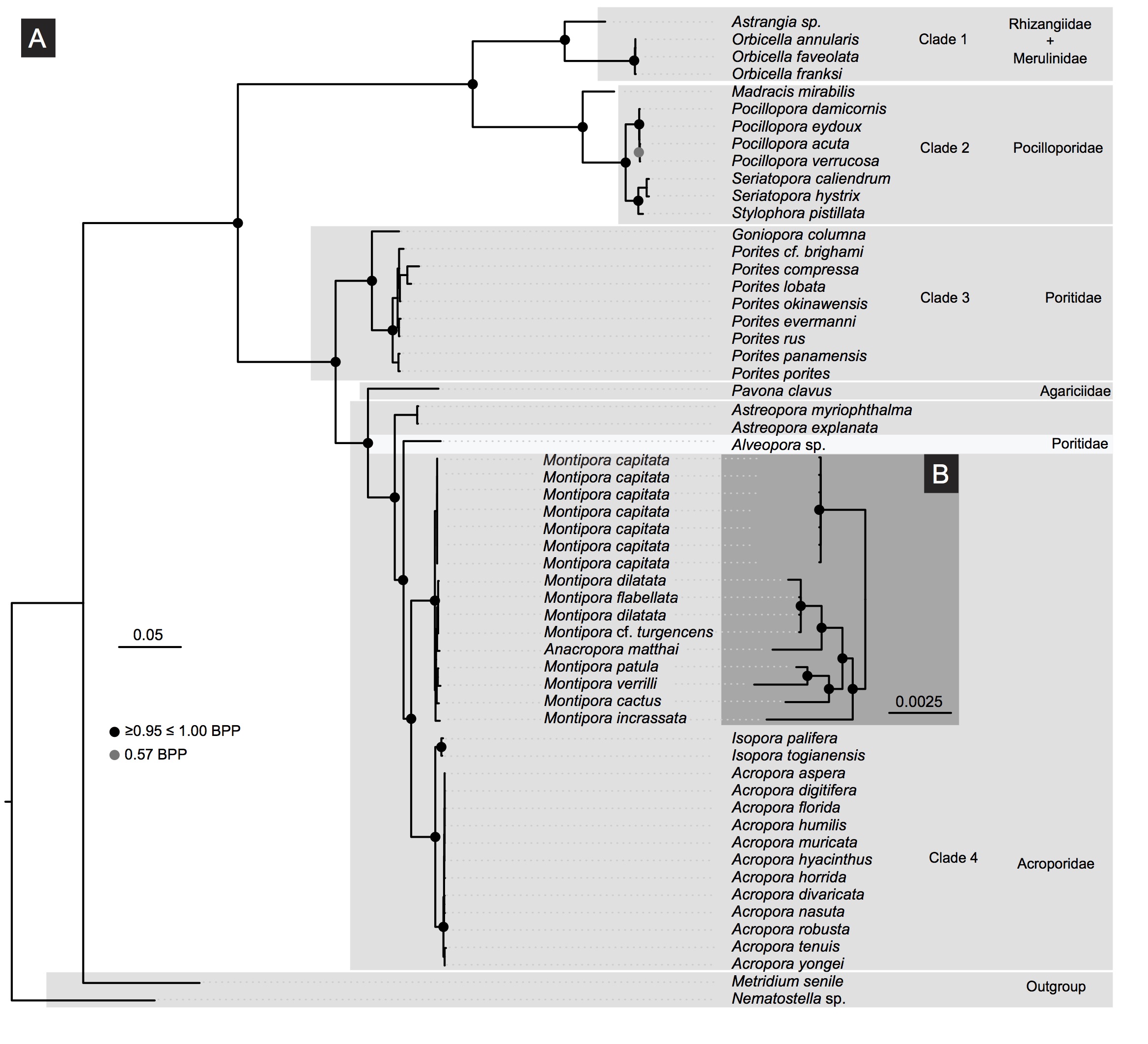

Supplement: Supplementary file 2 — S2. A. Bayesian analysis showing phylogenetic relationships among 55 scleractinian corals (49 species plus five morphotypes and pooled samples within M. capitata) representing 6 families and 15 genera plus the two outgroups Nematostella sp. and Metridium senile based on the concatenated mitochondrial dataset (13 protein-coding genes). B. Inset showing a detail of the topology referring to the genus Montipora zoomed 20x. This analysis was produced by MrBayes under a homogeneous model of rate change. Bayesian posterior probability (BPP) values are shown in black circles for values of maximal probability (1.00) and a grey circle for a value of 0.57. (JPG 587 kb) [file 12862_2019_1476_MOESM2_ESM.jpg]

A

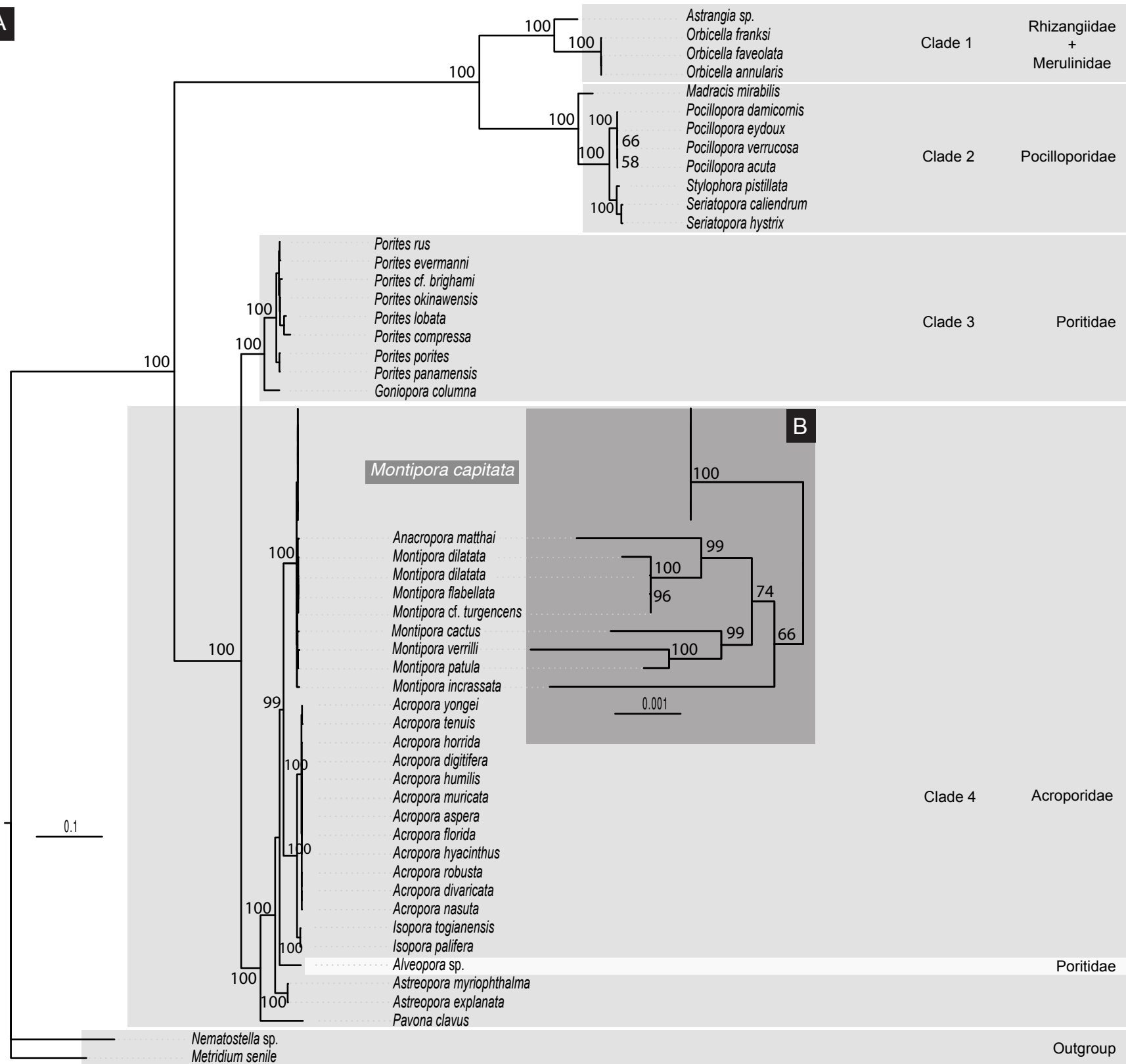

B

Supplement: Supplementary file 3 — S3. A. Maximum likelihood phylogram of 55 scleractinian corals (49 species plus five morphotypes and pooled samples within M. capitata) representing 6 families and 15 genera plus the two outgroups Nematostella sp. and Metridium senile based on the concatenated mitochondrial dataset (13 protein-coding genes) produced by RAxML under a gamma model of rate heterogeneity. B. Inset showing a detail of the topology referring to the genus Montipora zoomed 100x. Numbers at the nodes represent bootstrap proportions. (PDF 230 kb) [file 12862_2019_1476_MOESM3_ESM.pdf]

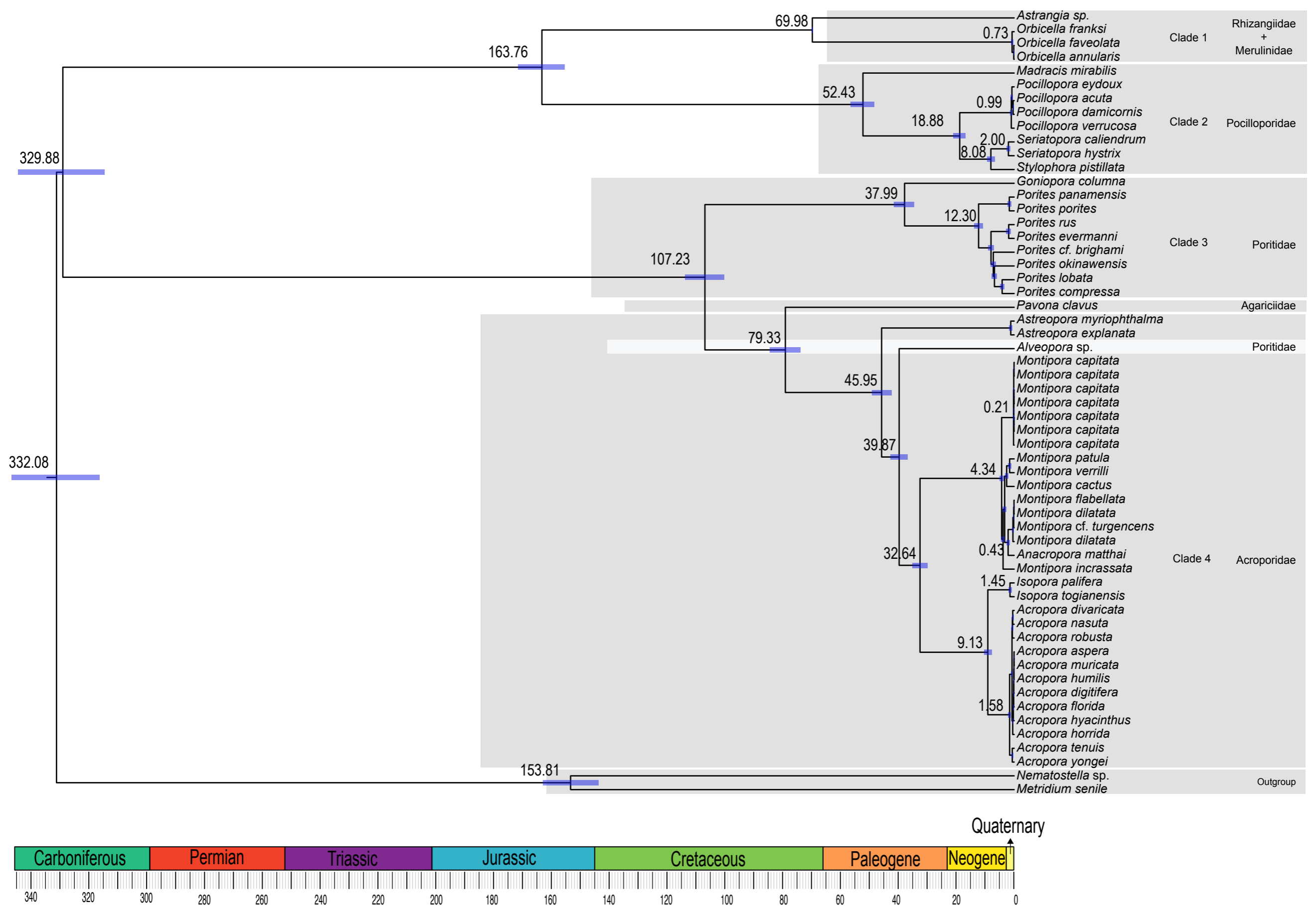

Supplement: Supplementary file 4 — S4. Beast maximum clade credibility chronogram showing main cladogenetic events among 55 scleractinian corals (49 species plus five morphotypes and pooled samples within M. capitata) representing 6 families and 15 genera plus the two outgroups Nematostella sp. and Metridium senile. The 95% highest posterior density (HPD) intervals are represented by the blue bars, and numbers at the nodes represent million years. (PDF 226 kb) [file 12862_2019_1476_MOESM4_ESM.pdf]

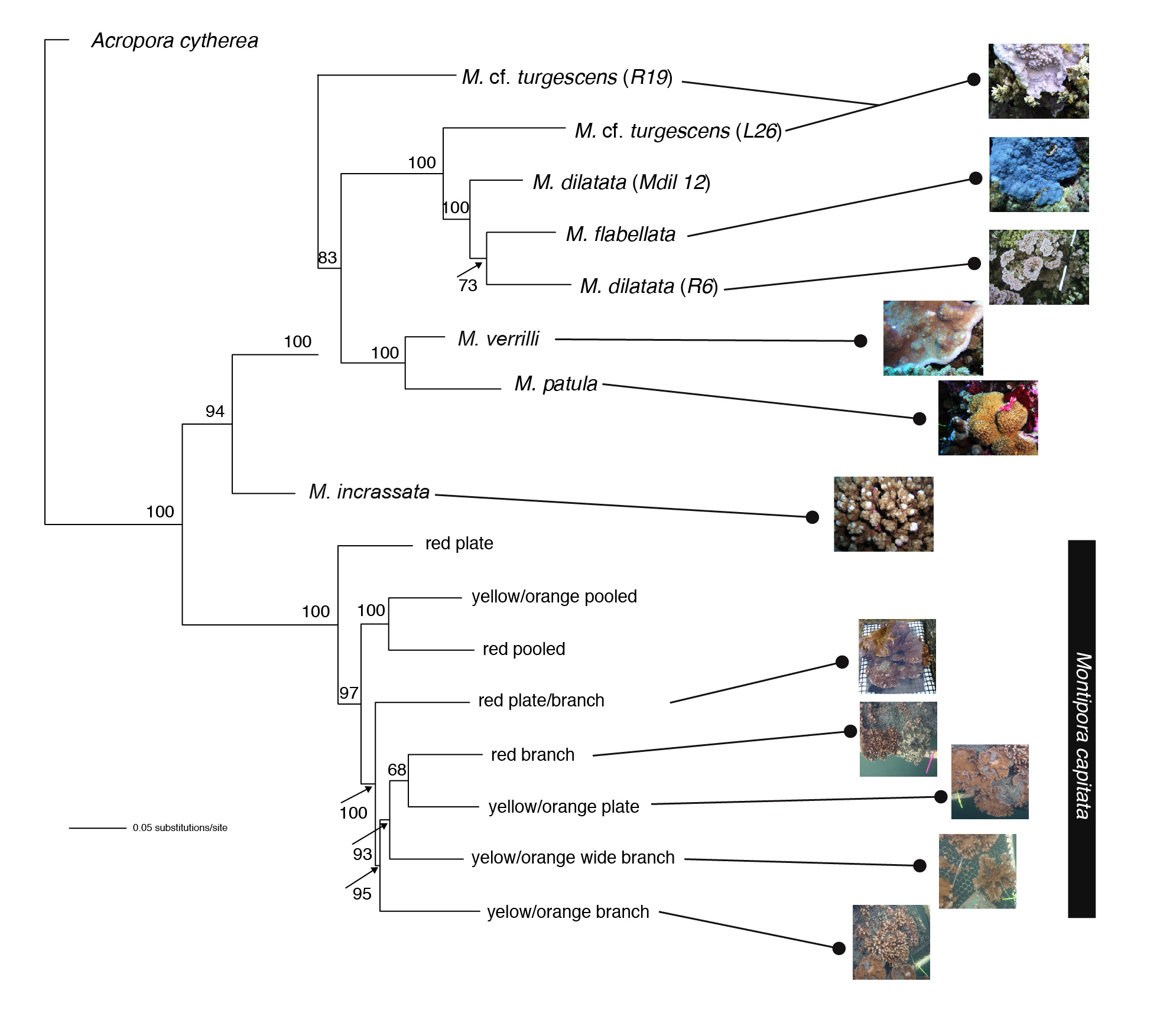

Supplement: Supplementary file 5 — S5. Species tree based on 60,602 SNPs from the ‘coral’ data set estimated with SVDquartets. Numbers at the nodes represent bootstrap proportions. Image plates represent in-situ photographs of Montipora species sampled for this study and of the morphotypes within Montipora capitata. (JPG 386 kb) [file 12862_2019_1476_MOESM5_ESM.jpg]

Total loci = 67,598

*Montipora capitata*  
43,423

*Symbiodinium*  
*minutum*  
4,286

*Fugacium*  
*kawagutii*  
4,727

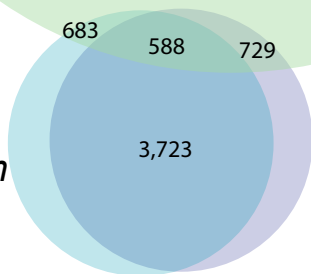

Supplement: Supplementary file 6 — S6. Venn diagram showing the overlap between putative coral (Montipora capitata) and symbionts (Symbiodinium minutum and Fugacium kawagutii) loci. (PDF 109 kb) [file 12862_2019_1476_MOESM6_ESM.pdf]

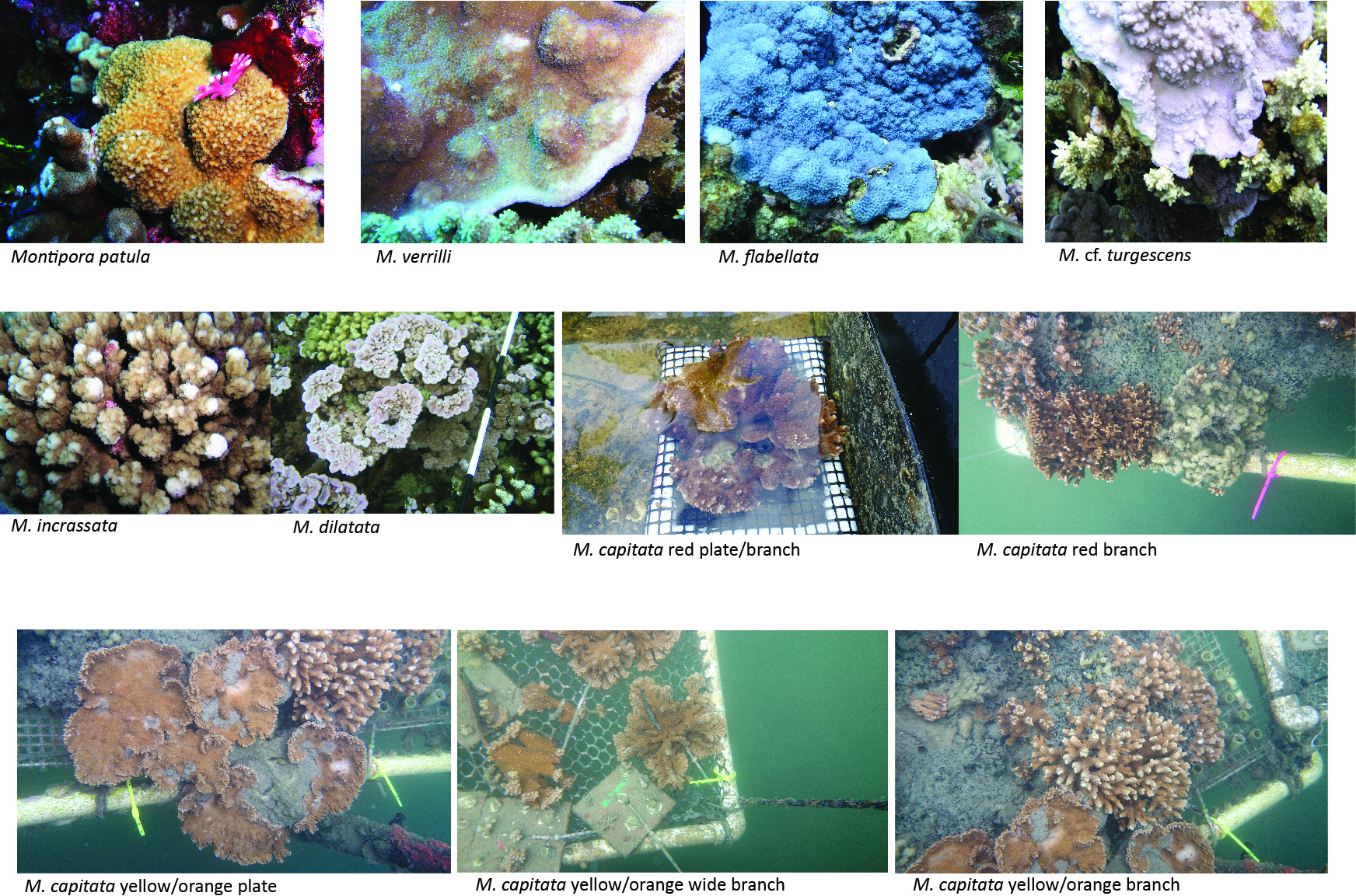

Supplement: Supplementary file 7 — S7. In-situ photographs of Montipora species sampled for this study and of the morphotypes within Montipora capitata. (JPG 3488 kb) [file 12862_2019_1476_MOESM7_ESM.jpg]
